# From Doping Intentions to Life Aspirations: A Goal Systems Perspective of Performance Enhancement in Sport

**DOI:** 10.3390/sports14060219

**Published:** 2026-05-26

**Authors:** Despoina Ourda, Lida Skoufa, Andreas Loukovitis, Haralambos Tsorbatzoudis, Vassilis Barkoukis

**Affiliations:** 1 Department of Physical Education and Sport Sciences, Aristotle University of Thessaloniki, 54124 Thessaloniki, Greece; lida.skoufa@yahoo.gr (L.S.); loukovit@phed.auth.gr (A.L.); lambo@phed.auth.gr (H.T.); bark@phed.auth.gr (V.B.); 2 Department of Health and Life Sciences, Frederick University, 1036 Lefkosia, Cyprus

**Keywords:** internal and external goals, Goal Systems Theory, Self-Determination Theory, motivational processes, nutritional supplements, competitive athletes

## Abstract

Doping research has predominantly focused on proximal cognitive predictors of athletes’ intentions to use prohibited substances, often conceptualizing doping as a final behavioral outcome. Drawing on Self-Determination Theory and Goal Systems Theory, the present study examined the relationships between doping intentions, perceived means of performance enhancement, and internal and external life aspirations among competitive athletes. A total of 204 athletes (Mage = 22.99 years) completed measures assessing doping intentions, perceived effectiveness of different performance enhancement means, and aspiration components. The results indicated that stronger doping intentions were negatively associated with internal aspiration components and positively associated with external aspiration components. Doping intentions were also positively related to perceived effectiveness of nutritional supplements, doping, and combined enhancement practices, while being negatively associated with reliance on training and nutrition alone. Several indirect effects were observed, demonstrating that perceived performance enhancement partially mediated the relationships between doping intentions and aspiration components. These findings suggest that performance enhancement behaviors are cognitively embedded within athletes’ motivational goal systems and play an active role in shaping aspiration-related evaluations. Overall, this study advances doping research by conceptualizing doping behavior as part of a broader, goal-directed, motivational structure rather than an isolated outcome.

## 1. Introduction

Participation in sport has been consistently associated with a wide range of physical, psychological, and social benefits, including improved health, well-being, and quality of life [1,2]. However, alongside its positive outcomes, sport also encompasses behaviors and pressures that can undermine its values and intended benefits. Among the most concerning manifestations of this “dark side” is the use of doping substances, which represents one of the most serious challenges to the integrity of sport and poses significant health risks for athletes [3,4]. Consequently, research has increasingly focused not only on the prevalence of doping but also on the psychological and social mechanisms that underlie athletes’ decisions to engage in such behaviors [5,6].

Social–cognitive models have been particularly influential in explaining doping-related behaviors. Among these, the Theory of Planned Behavior [7] has been widely applied to predict athletes’ intentions to use prohibited substances [5,6]. Consistent with this model, attitudes toward doping, perceived social norms, and perceived behavioral control have all been found to predict doping intentions across competitive and recreational settings [8,9,10]. Other factors, such as moral disengagement, self-regulatory efficacy, situational temptation, and past doping use, have also emerged as significant predictors [10].

While this model has greatly enhanced our understanding of the proximal cognitive mechanisms underlying doping, it primarily conceptualizes doping as an end in itself, a final behavioral outcome to be explained. Yet, evidence suggests that doping may instead function as a means to attain more highly valued ends, such as winning, improving performance, or fulfilling fundamental psychological needs [11,12,13,14]. In this sense, athletes’ engagement in doping-related behaviors might be better understood within a broader motivational system in which sport behaviors serve higher-order goals and aspirations.

From the perspective of Self-Determination Theory (SDT), aspirations represent enduring life goals that individuals consider essential for their well-being [15]. These aspirations may be intrinsic, such as health, personal growth, and community contribution, or extrinsic, such as wealth, fame, and image. This distinction between internal (intrinsic) and external (extrinsic) aspirations has been widely applied in sport psychology to examine how athletes’ overarching life goals relate to their motivation and well-being [16]. Sport participation provides a meaningful context through which individuals pursue both types of aspirations [17]. Athletes driven by intrinsic aspirations typically experience greater vitality, satisfaction, and self-determined motivation, whereas those primarily oriented toward extrinsic aspirations (e.g., appearance, recognition) are more likely to exhibit controlled motivation and burnout [17,18] and may be associated with a greater tendency to use performance-enhancing substances [5,6].

Empirical evidence suggests that athletes’ life aspirations are shaped by contextual and cultural influences within the sport environment, including coaching behaviors, sport type, and prevailing motivational climates [17,19]. In line with Self-Determination Theory, autonomy-supportive environments foster psychological need satisfaction and autonomous functioning, whereas controlling climates promote need frustration and diminished well-being [15,20,21]. According to goal content mini-theory, environments that support basic psychological needs are also more likely to encourage intrinsic aspirations (e.g., personal growth, health), whereas controlling contexts may reinforce extrinsic aspirations (e.g., fame, image; [17]).

Within this motivational framework, sport-related behaviors, such as training practices, nutritional strategies, and the use of dietary supplements, may function as instrumental means through which athletes attempt to attain their higher-order aspirations. When athletes define success in external, normative terms, performance-enhancing practices may be perceived as legitimate or necessary tools for achieving externally defined success, status, or appearance-related goals [22,23]. This instrumentalization of behavior may extend along a continuum from legal nutritional supplementation to more ethically problematic practices, including doping, to achieve these externally oriented goals [12,13,20]. Conversely, athletes oriented toward intrinsic aspirations, such as health, mastery, and long-term development, are more likely to adopt nutrition and supplementation behaviors aligned with well-being and ethical sport participation and reject doping as incompatible with their core values and self-endorsed goals [10,24]. Collectively, this evidence suggests that athletes’ aspirations may represent an important motivational pathway linking the sport environment to decisions regarding nutritional supplement use and doping-related attitudes and intentions. From this perspective, doping may not represent an ultimate objective but rather one of several perceived pathways toward broader goals such as success, recognition, or financial stability [12,13,14]. Understanding doping as a goal-directed behavior embedded within athletes’ broader motivational structures allows researchers to move beyond the intention-based focus of social–cognitive models and consider how specific behaviors are cognitively organized around the pursuit of valued ends. To address this conceptual gap, the Goal Systems Theory (GST; [25,26,27]) provides a comprehensive framework for linking proximal behavioral intentions with distal life aspirations, conceptualizing behaviors as dynamic means that serve higher-order goals.

The GST conceptualizes motivation as a cognitive network of interrelated goals and means. Goals are represented as mental structures connected to the behaviors, strategies, and resources that can help achieve them, while these means may also be linked to one another. A single goal can be pursued through multiple means (e.g., training, diet, supplementation), and one means can serve multiple goals (e.g., enhance performance to gain recognition or financial success). The strength of these cognitive links depends on the number and perceived importance of the available means: when few options exist to achieve a valued goal, the existing links become stronger and more likely to guide behavior [28].

Individuals are more likely to invest resources in means that they perceive as both effective and necessary for attaining their higher-order goals [28]. Within the sport context, the GST may provide a dynamic framework for understanding how intentions toward specific behaviors, such as training practices, supplement use, or doping, could be cognitively organized within broader goal systems that reflect athletes’ aspirations and personal values. Doping intentions, in particular, are theoretically expected to be associated with life aspirations because they are not formed in isolation but are embedded within individuals’ broader motivational and goal systems [23,29]. From a Self-Determination Theory perspective, life aspirations reflect enduring representations of what individuals consider important and worthwhile for their well-being [15]. Behaviors such as doping may be cognitively evaluated in terms of their perceived compatibility with these higher-order goals. When athletes prioritize external aspirations and define success in normative, outcome-based terms, doping may be construed as an effective or necessary means for attaining these valued ends. Conversely, athletes who strongly endorse internal aspirations are more likely to perceive doping as incompatible with their core values and long-term well-being. Within this framework, doping intentions reflect the strength of the cognitive association between performance-enhancing means and distal life aspirations. Accordingly, variation in athletes’ aspiration content should be systematically related to their willingness to engage in doping-related behaviors, as intentions emerge from the perceived alignment between specific actions and the pursuit of personally meaningful life goals [15]. This conceptualization might be important in better understanding doping behavior in sport, as previous theorizing and evidence suggested that doping may be a means of achieving higher-order goals such as improved performance, wealth, or fame [30,31].

The integration of SDT and GST provides added conceptual value because the two theories address different levels of the motivational process. SDT explains the content and quality of athletes’ higher-order aspirations, distinguishing between internal aspirations, such as health and personal growth, and external aspirations, such as fame, image, and wealth. GST extends this account by explaining how these aspirations become cognitively connected to specific means of goal attainment. From this perspective, training and nutrition, supplement use, and doping may represent different perceived pathways through which athletes attempt to achieve valued goals. Therefore, GST adds explanatory value beyond SDT by specifying the mechanisms through which aspiration content may become linked to specific doping-related cognitions and performance enhancement choices. Still, research investigating such goal systems with respect to doping is scarce. Skoufa et al. [31] confirmed that several performance enhancement practices are associated with the decision-making process toward the use of performance enhancement substances. However, there is currently no empirical evidence clarifying how means of performance enhancement (e.g., nutritional supplements and doping) operate within the relationship between doping intentions and life aspirations. In particular, it remains unclear whether and how these means are integrated into the formation of aspirations (e.g., internal versus external) and how they shape the underlying motivational and decision-making processes related to doping intentions. The present study set out to address this gap and investigate the mediating role of performance enhancement means in the relationship between doping intentions and life aspirations. Therefore, the present study integrates these two conceptualizations; it not only examines whether aspiration content is associated with doping intentions but also whether perceived means of sport success partly explain these associations. This integration helps conceptualize doping not as an isolated behavioral outcome but as one possible means within a broader, goal-directed, motivational system. Based on previous theorizing, we hypothesized that (a) doping intention would be negatively associated with internal aspiration components and positively associated with external aspiration components (H1); (b) doping intention would be positively associated with the use of performance-enhancing means, which include nutritional supplements and/or doping, and negatively associated with use of performance-enhancing means, such as training and nutrition alone (H2); and (c) means of performance enhancement would mediate the relationship between doping intention and internal and external aspiration components (H3).

## 2. Materials and Methods

### 2.1. Participants

A total of 204 athletes participated in this study. The sample consisted of 122 males (59.8%) and 82 females (40.2%), with a mean age of 22.99 years (SD = 5.41). Participants were actively involved in competitive sports (M years of involvement = 10.23 years, SD = 4.43), representing sports such as athletics, handball, and basketball. Eligible athletes were training regularly and competing at regional and national levels. Given the eligibility criteria for participation, a convenience sampling approach was employed. Specifically, sports clubs served as the units of selection and were invited to participate based on accessibility and willingness to collaborate. Athletes who met the inclusion criteria and were affiliated with the participating clubs were subsequently recruited for this study.

### 2.2. Measures

Doping intentions: Athletes’ intentions to use prohibited substances were assessed with the relevant items used in previous research conducted in Greece [11,32]. The instrument consisted of three items rated on a 7-point Likert scale (e.g., 1 = strongly disagree to 7 = strongly agree), which captured the extent to which athletes expressed willingness or readiness to engage in doping practices in the future. Higher scores reflected stronger intentions to use banned substances. Previous research supports the reliability and construct validity of the scale in athlete populations. In the present study, internal consistency was satisfactory (Cronbach’s α = 0.95).

Means of achieving sport success: Athletes’ perceptions of the strategies that can help them achieve success in sport were examined via six items. Participants responded to the stem question, “How much can the following help you achieve your goals in sport?”, followed by six different pathways to athletic achievement: training and nutrition, nutritional supplements, doping, training and nutrition AND nutritional supplements, training and nutrition AND doping, and nutritional supplement AND doping. Responses were given on a 7-point Likert scale ranging from 1 (not at all) to 7 (very much), with higher scores reflecting a stronger belief in the effectiveness of the respective means. Each item of the subscale was treated as a separate means for achieving success [31]. The use of single-item indicators was guided by the goal systems framework, in which specific means could be represented as discrete pathways linked to higher-order goals. In the present study, the aim was not to assess broad latent constructs of performance enhancement but to examine athletes’ perceived instrumentality of clearly distinguishable means to sport success, such as training and nutrition, nutritional supplements, doping, and combined enhancement practices. Therefore, each item was treated as a separate perceived means.

Athletes’ life aspirations: Life aspirations were assessed with a shortened version of the Aspiration Index [33]. This instrument consisted of seven subscales that reflected either intrinsic or extrinsic life aspirations. The subscales that indicated extrinsic life aspirations were fame, wealth, and image. In contrast, the subscales personal growth, relationships, community, and health indicated intrinsic life aspirations. In the present study, the dimensions of relationships and community were not measured as they were deemed irrelevant to the context of doping. Each of these subscales consisted of five items. All items directly captured the essence of intrinsic or extrinsic life aspirations. Participants evaluated each aspiration on three dimensions: (1) how important the goal is to them personally, (2) how likely they believe it is that they will achieve it, and (3) the extent to which they feel they have already achieved it. Responses were anchored on a 7-point Likert scale ranging from 1 (not at all) to 7 (very much).

### 2.3. Procedure

This study adhered to the ethical standards for research of the Declaration of Helsinki and obtained approval from the Ethics in Research Committee of the Department of Physical Education and Sport Science of the Aristotle University of Thessaloniki (protocol number 21/2021). Recruitment was carried out through contacts with sports clubs and federations. Official approval for recruitment was first achieved by the relevant sports clubs and governing bodies. Coaches and team administrators were then informed about the aims and procedures of this study, and they assisted in communicating the study details to athletes and, where necessary, their parents. Participation was voluntary and all athletes provided informed consent to take part in this study before data collection. For those under the age of 18, consent was additionally obtained from their parents or legal guardians. Athletes completed the survey anonymously in a paper-and-pencil format, either during scheduled team sessions or in their own time, with coaches instructed to emphasize the voluntary nature of participation and the confidentiality of responses. On average, the completion time was approximately 20 min.

### 2.4. Data Analysis

All statistical analyses were conducted using SPSS Version 29 and Mplus 8.0. Descriptive statistics and bivariate correlations were obtained with SPSS, while the hypothesized model was tested through path analysis in Mplus. Given the modest sample size, the number of estimated parameters, and the inclusion of several distinct performance enhancement means, the full structural equation modeling (SEM) model could not be tested. Thus, path analysis was employed to address this study’s focus on testing theoretically specified directional relationships among observed variables (i.e., means). All variables included in the model were composite scores derived from previously validated measures, reducing the need to model measurement error explicitly. Given the relatively modest sample size (N = 204) and the complexity that would have been introduced by specifying a full measurement model, path analysis offered a more parsimonious and statistically appropriate approach. This analytic strategy allowed for the simultaneous estimation of direct and indirect effects, facilitating the examination of mediation pathways linking doping intention, means of performance enhancement, and internal and external aspiration components, while maintaining adequate statistical power and model stability. Model fit was evaluated with several commonly recommended indices: the Comparative Fit Index (CFI), the Root Mean Square Error of Approximation (RMSEA), and the Standardized Root Mean Square Residual (SRMR). In line with conventional cutoffs, CFI values approaching or exceeding 0.90 were interpreted as evidence of acceptable fit, whereas RMSEA and SRMR values below 0.08 were taken to indicate satisfactory model fit [34].

## 3. Results

### 3.1. Descriptive Statistics

The sample consisted of 204 participants, with a mean age of 22.99 years (SD = 5.42). Of these participants, 122 (59.8%) were categorized as males and 82 (40.2%) as females. In terms of sports participation, basketball was the most frequently reported sport (28.9%), followed by track and field (21.6%) and handball (10.8%). Other notable sports included volleyball (7.4%), swimming (7.8%), and Muay Thai (7.4%). Less common sports, such as rowing, were reported by fewer participants, each accounting for 1.0% to 3.0% of the sample. An ANOVA revealed no statistically significant differences between team and individual sports in the vast majority of the study variables. Regarding training experience, the mean years of training was 10.23 (SD = 4.432). The most frequently reported durations of training were 10 years (10.8%), 12 years (10.8%), and 15 years (10.3%). Other notable durations included 7 years (8.8%), 8 years (8.3%), and 9 years (9.8%). The mean scores and analysis of correlation among the study variables are presented in Table 1. The analysis of correlations revealed mainly low to moderate associations among the variables. Intention was positively associated with the use of nutritional supplements, doping, and combined behavioral patterns involving supplements and doping, while it was negatively related to training and nutrition, as well as to all internal regulation dimensions (importance, likelihood, and attainment). In contrast, training and nutrition showed consistent positive correlations with internal importance and internal likelihood and negative associations with doping-related behaviors. Strong associations were observed among doping-related variables, particularly between training and nutrition and doping (r = 0.82), indicating substantial overlap among these behaviors. Regarding aspirations, internal dimensions were strongly interrelated (rs = 0.32–0.67) and generally negatively associated with doping-related means, whereas external dimensions were strongly correlated with each other (rs = 0.72–0.83) and showed positive associations with intention, supplement use, and doping.

### 3.2. Model Testing

A structural path analysis was tested using Mplus 8.10 to examine the direct and indirect effects of intention on six outcome variables representing internal and external motivation components via means. The model was estimated using maximum likelihood (ML), with data from 204 participants. Overall, model fit was mixed. The Comparative Fit Index (CFI) indicated excellent fit (CFI = 0.975). The Root Mean Square Error of Approximation (RMSEA) was high at 0.164 (90% CI [0.121, 0.211]), and the chi-square test was significant (χ^2^(7) = 45.50, *p* < 0.001), suggesting some misspecification. The Standardized Root Mean Square Residual (SRMR) was acceptable at 0.069. Despite the elevated RMSEA, the CFI and SRMR met conventional thresholds for acceptable fit, indicating that the model provided an adequate representation of the data. This discrepancy is consistent with previous findings showing that RMSEA can overestimate lack of fit in models with small degrees of freedom (df = 8), rendering it less reliable in such cases [35]. Although RMSEA may be inflated in models with low degrees of freedom, this value nevertheless suggests that the model should be interpreted cautiously. Therefore, the findings are presented as theoretically informed associations among the study variables rather than as definitive evidence of a fully fitting structural model. The model was retained because it was directly derived from the hypotheses and because theoretically plausible alternative specifications did not yield a more stable or interpretable solution.

Direct paths revealed significant associations of intention with internal versus external factors. Specifically, intention was negatively related to internal importance (β = −0.19, *p* = 0.007), internal likelihood (β = −0.35, *p* < 0.001), and internal attainment (β = −0.35, *p* < 0.001). In contrast, intention was positively associated with external importance (β = 0.25, *p* < 0.001), external likelihood (β = 0.17, *p* = 0.015), and external attainment (β = 0.25, *p* < 0.001). In addition, intention significantly predicted several means variables, with positive associations observed for nutritional supplements (β = 0.33, *p* < 0.001); doping (β = 0.30, *p* < 0.001); training, nutrition, and nutritional supplements (β = 0.23, *p* = 0.001); and training, nutrition, and doping (β = 0.26, *p* < 0.001). A negative relationship was found for training and nutrition (β = −0.19, *p* = 0.005), whereas the association between nutritional supplements and doping was nonsignificant.

**Figure 1:**
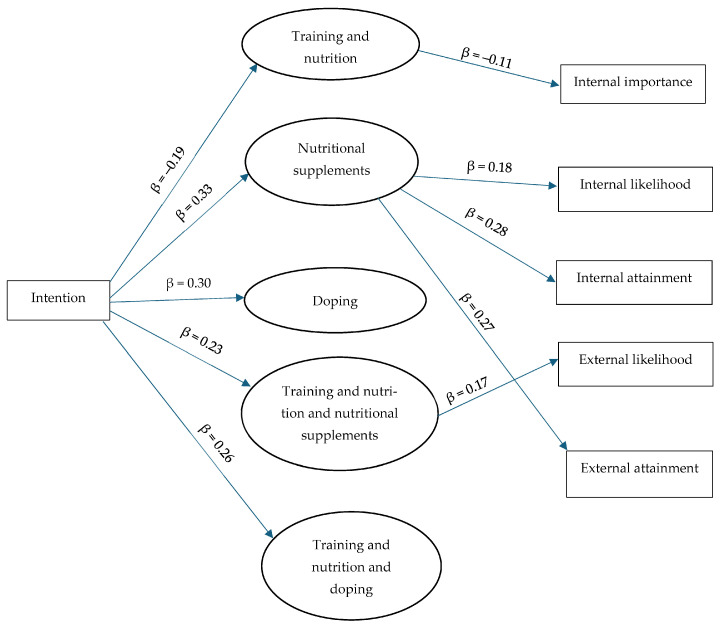
Structural paths among the study variables.

Some specific indirect effects via the means variables were significant. For example, the association from intention to internal importance via training and nutrition was negative and significant (β = −0.111, *p* = 0.023), while intention to internal likelihood via nutritional supplements (β = 0.188, *p* = 0.004) and intention to internal attainment via nutritional supplements (β = 0.283, *p* = 0.001) were positive. Total indirect effects were significant for internal importance (β = −0.146, *p* = 0.050), internal attainment (β = 0.254, *p* = 0.001), external likelihood (β = 0.310, *p* = 0.028), and external attainment (β = 0.293, *p* = 0.016), indicating partial mediation (Figure 1). Due to the high correlation between the means of training and nutrition and doping, we ran multicollinearity diagnostics. The results indicated that the highest VIF values were 3.35 and 3.96, with corresponding tolerance values of 0.29 and 0.25. Although these values suggest moderate overlap among predictors, they are below conventional cut-off values for severe multicollinearity. Sensitivity analyses excluding each of the highly correlated predictors in turn produced a substantively similar pattern of results, supporting the robustness of the findings.

## 4. Discussion

The purpose of the present study was to examine doping intentions and performance enhancement behaviors not merely as proximal behavioral outcomes but as elements embedded within a broader motivational goal system related to life aspirations. Drawing on Self-Determination Theory and Goal Systems Theory, the study investigated whether athletes’ intentions to use doping substances were directly associated with internal and external life aspirations and whether this relationship was mediated by different perceived performance enhancement means. Overall, the findings support the conceptualization of doping as an instrumental means rather than an isolated endpoint. Doping intention was consistently associated with lower endorsement of internal aspirations and stronger endorsement of external aspirations, while also predicting greater reliance on supplement use and doping-related means and reduced reliance on training and nutrition alone. Importantly, several of these means partially mediated the relationship between intention and aspiration components, indicating that athletes’ perceptions of how success can be achieved play a meaningful role in shaping their aspiration-related beliefs.

### 4.1. Self-Determination and Goal Systems Integration

The findings related to the first hypothesis largely support the proposed associations between doping intention and athletes’ life aspirations. The findings suggest that athletes who express a greater willingness to use prohibited substances place less value on intrinsically oriented life goals such as health and personal growth and are more strongly oriented toward extrinsic goals, including wealth, fame, and image. These results are in line with Self-Determination Theory [15] and reinforce the relevance of aspiration content in understanding doping-related motivation. Importantly, these results are consistent with previous empirical evidence indicating that extrinsic forms of motivational regulations are associated with maladaptive outcomes in sport, including positive attitudes toward doping [5,6,36], whereas athletes who prioritize intrinsic aspirations such as health and personal growth tend to report lower susceptibility to doping-related temptations [10]. From a doping prevention perspective, these findings suggest that interventions may be more effective when they address athletes’ underlying definitions of success and life goals. Promoting intrinsic aspirations may reduce the motivational compatibility between doping intentions and externally defined success, thereby contributing to more sustainable and value-consistent anti-doping strategies.

The present findings offer partial support for the second hypothesis. Our findings align with integrative theoretical perspectives that emphasize the role of goal-means alignment in determining the motivational potency of specific behaviors: when a means is perceived as instrumentally valuable to a highly valued goal (e.g., performance enhancement), individuals are more likely to adopt those means [31]. Previous research has similarly observed that nutritional supplement use and doping-related behaviors often co-occur and share underlying motivational drivers, such as extrinsic orientation or performance emphasis, even if the behaviors themselves differ in legality or risk (see [22,37]). Our findings suggest that nutritional supplements and doping-related means may share motivational correlates and be perceived as instrumentally relevant to sport success among athletes with stronger doping intentions. Still, the differential associations with means suggest that not all performance-related behaviors are equivalently associated with the motivational architecture of athletes: health-oriented practices (training and nutrition) may be associated with higher autonomous motivation, whereas supplement and doping-related practices may be associated with controlled forms of motivation that align with doping intentions. This supports arguments from both GST and SDT that the cognitive representation of means and their perceived instrumentality are associated with behavioral intentions and decision-making [31]. Moreover, these results challenge simplistic interpretations of supplement use as merely a gateway to doping. Doping intentions were associated with both nutritional supplements and doping-oriented means, suggesting that they may co-occur as part of a broader pattern of a performance-enhancing mindset associated with shared underlying goals and values (co-occurrence hypothesis, [22]). For prevention, interventions should target how athletes interpret the role and instrumentality of legal enhancement practices (e.g., supplements, training strategies) in relation to their aspirations to help decouple performance goals from doping-oriented means and promote engagement with healthy, autonomous performance-enhancing behaviors.

The findings provide partial but meaningful support for the third hypothesis. Our findings align with GST, which posits that behaviors are cognitively represented as means that connect proximal intentions to more distal goals and that the perceived effectiveness of these means is associated with goal commitment and goal-related cognitions [25]. Although the found indirect associations are theoretical representations rather than evidence of temporal or causal sequences, they highlight that means are not motivationally neutral but are actively associated with the structure of athletes’ goal systems. In line with recent evidence by Skoufa et al. [31], performance-enhancing practices appear to function as interconnected means that are associated with decision-making processes related to doping, rather than serving merely as behavioral outcomes. The present findings extend this work by showing that such means also contribute to how athletes cognitively engage with their life aspirations, suggesting that athletes who perceive performance enhancement practices as necessary or instrumental for success may integrate these behaviors into broader success-oriented goal structures, increasing the motivational compatibility between doping intentions and extrinsically defined aspirations [30]. These findings underscore the importance of targeting athletes’ beliefs about the necessity and effectiveness of different performance-enhancing means, rather than focusing exclusively on intentions or attitudes toward doping itself.

### 4.2. Limitations and Strengths

Several limitations of the present study should be acknowledged when interpreting the findings. First, the cross-sectional design precludes any causal inferences regarding the directionality of the relationships among doping intentions, perceived means of performance enhancement, and life aspirations. Although the proposed model was theoretically grounded in SDT and GST, it is equally plausible that aspiration orientations shape perceptions of means or even doping intentions over time [29]. Longitudinal or experimental designs are therefore needed to clarify temporal ordering and causal mechanisms within athletes’ motivational goal systems. Secondly, all variables were assessed using self-report measures, which may be vulnerable to social desirability bias and response distortion, particularly because doping-related intentions concern a sensitive and socially sanctioned behavior. Although anonymity and confidentiality were assured to reduce evaluation concerns, these safeguards cannot fully rule out the possibility that athletes underreported their willingness to use prohibited substances or aligned their responses with socially desirable anti-doping norms. As a result, the observed associations involving doping intentions may represent conservative estimates. Future studies should include social desirability scales, randomized response or indirect questioning techniques, behavioral indicators, and implicit assessments to better control for response bias and strengthen the validity of doping-related measurement. Moreover, this study relied on a convenience sample of competitive athletes, which may limit the generalizability of the findings to other populations, such as elite athletes, recreational exercisers, or adolescents in early sport specialization stages. Differences in competitive level, sport type, and cultural context may influence how aspirations and performance-enhancing means are cognitively organized. Future studies with larger and more balanced samples should examine sport type and competitive level as covariates, moderators, or grouping variables and test whether the proposed goal system relationships differ across individual and team sports, regional and national competition levels, and stages of athletic development. Additionally, the means of performance enhancement were measured using single-item indicators, which, while theoretically justified within a goal systems framework, do not allow estimation of internal consistency and may be more vulnerable to measurement error than multi-item scales. Future studies should incorporate validated, multi-item scales of perceived performance enhancement means, preferably analyzed through latent variable models that account for measurement error. Finally, although path analysis was selected as a parsimonious approach suitable for the sample size and research aims, several constructs in the model, particularly aspirations, are theoretically latent. By using composite scores, the analysis did not model measurement error explicitly, which may have biased parameter estimates or affected the strength of the observed associations. A full SEM approach would have been preferable; however, attempts to estimate a latent variable model did not converge, most likely due to the relatively modest sample size and the number of parameters required. In addition, model fit was mixed: although CFI and SRMR suggested acceptable fit, the RMSEA was substantially elevated, indicating possible model misspecification. Therefore, the findings should be interpreted with caution and viewed as preliminary evidence for the proposed goal system relationships. Future studies with larger samples should test the model using full SEM, compare theoretically competing models, report additional fit indices, and examine whether longitudinal or network-based approaches provide a more precise representation of the relationships among doping intentions, perceived means of performance enhancement, and life aspirations.

Despite these limitations, the present study contributes to the doping literature by advancing a goal system perspective on doping intentions, situating them within athletes’ broader motivational and aspirational frameworks. The findings provide overall support for the proposed goal systems perspective. Doping intentions were negatively associated with internal aspirations and positively associated with external aspirations, supporting H1. They were also positively related to the perceived effectiveness of nutritional supplements, doping, and combined enhancement practices and negatively related to reliance on training and nutrition alone, supporting H2. H3 was partially supported, as several, but not all, indirect effects through perceived performance enhancement means were significant. The findings also illustrate the added value of integrating SDT and GST. SDT helps explain why aspiration content matters: athletes who prioritize internal aspirations may evaluate doping as inconsistent with health, personal growth, and long-term development, whereas athletes who prioritize external aspirations may evaluate doping-related means as more compatible with externally defined success. GST extends this interpretation by showing that these aspirations are not linked to doping intentions in isolation but through athletes’ beliefs about which means are effective for achieving sport success. In this sense, the goal–means structure provides an additional explanatory layer beyond aspiration content alone by identifying the perceived performance enhancement pathways through which broader life goals may become connected to doping-related cognitions. Rather than conceptualizing doping intention as an isolated or purely proximal outcome, the findings demonstrate that it is systematically linked to athletes’ life aspirations and their perceived means of achieving sport success. By integrating GST with SDT, this study shows that performance-enhancing behaviors function as theoretically proposed instrumental pathways that connect intentions to higher-order goals, reinforcing certain motivational orientations while undermining others.

### 4.3. Practical Implications

From a practical standpoint, these findings suggest that doping prevention interventions should move beyond information provision and address how athletes define success and which means they perceive as necessary for achieving it. Coach education programs could promote autonomy-supportive practices, such as involving athletes in goal setting, providing meaningful rationales for training demands, acknowledging performance pressure, and reinforcing effort, learning, health, and long-term development rather than only winning or external recognition. Such practices may strengthen intrinsic aspirations and reduce the perceived need to rely on risky enhancement strategies. In addition, values-based anti-doping education supporting the spirit of sport values and fair play could help athletes reflect on the type of athlete and person they want to become and on whether doping is compatible with their life goals, such as health, personal growth, integrity, and sustainable sport participation. Interventions could also include cognitive reframing of performance goals, helping athletes reinterpret success in terms of mastery, competence, resilience, and long-term development rather than fame, image, wealth, or recognition. Finally, prevention initiatives should target not only athletes but also coaches, parents, clubs, and athlete support personnel. Such educational efforts should assist them in supporting the psychological empowerment of athletes through the development of a broader sport climate supporting their autonomy, competence, relatedness, health, and personal development while weakening the perceived instrumental value of doping. Overall, this study underscores the value of adopting motivationally informed, systems-based approaches to understanding and preventing doping behavior in sport.

## Figures and Tables

**Table 1:** Descriptive statistics and correlations among the study variables.

	1	2	3	4	5	6	7	8	9	10	11	12	13
1. Intention	-												
2. Training and nutrition	−0.19	-											
3. Nutritional supplements	0.33	0.05	-										
4. Doping	0.30	−0.23	0.42	-									
5. Training and nutrition and supplements	0.22	0.13	0.66	0.38	-								
6. Training and nutrition and doping	0.26	−0.18	0.39	0.82	0.43	-							
7. Supplements and doping	0.08	−0.05	0.20	0.32	0.25	0.52	-						
8. Internal importance	−0.26	0.33	−0.06	−0.19	−0.03	−0.19	−0.04	-					
9. Internal likelihood	−0.35	0.30	0.07	−0.20	−0.02	−0.19	−0.07	0.67	-				
10. Internal attainment	−0.22	0.02	0.22	0.08	0.04	0.03	−0.04	0.32	0.67	-			
11. External importance	0.32	−0.02	0.30	0.18	0.24	0.14	0.12	0.25	0.19	0.16	-		
12. External likelihood	0.25	−0.03	0.32	0.12	0.29	0.07	0.07	0.14	0.36	0.32	0.83	-	
13. External attainment	0.37	−0.14	0.37	0.21	0.25	0.14	0.05	−0.06	0.11	0.36	0.72	0.82	-
Mean	5.41	6.37	4.39	3.21	5.70	3.93	4.38	62.17	56.62	50.52	65.06	63.67	57.19
SD	4.21	0.96	1.55	2.39	1.63	2.55	5.28	8.80	8.04	8.38	17.24	16.23	16.24

Note: Scores below 0.13 were non-significant (*p* > 0.05), scores between 0.14 and 0.18 were significant at the 0.05 level (*p* < 0.05), and scores above 0.19 were significant at the 0.01 level (*p* < 0.01).

## Data Availability

The raw data supporting the conclusions of this article will be made available by the authors upon request.
